# Optimistic Attributional Style as a Predictor of Well-Being: Exploring the Mediating Roles of Gratitude and Savoring the Moment

**DOI:** 10.11621/pir.2021.0304

**Published:** 2021-09-30

**Authors:** Victoria A. Titova Grandchamp, Tamara O. Gordeeva, Oleg A. Sychev

**Affiliations:** a Faculty of Psychology, Lomonosov Moscow State University, Moscow, Russia; b Higher School of Economics, International Laboratory of Positive Psychology of Personality and Motivation, Moscow, Russia; c Shukshin Altai State University for Humanities and Pedagogy, Russia

**Keywords:** Optimistic attributional style (OAS), subjective well-being (SWB), savoring the moment, gratitude, life satisfaction, happiness, dispositional optimism

## Abstract

**Background:**

The construct of attributional style refers to the specific ways people explain events, both positive and negative. An optimistic attributional style (OAS) for negative events has been shown to be reliably associated with low rates of depression (Peterson et al., 1985; Sweeney et al., 1986; Hu et al., 2015). On the contrary, an optimistic attributional style for positive events is a separate phenomenon associated mainly with well-being, but these relationships remain underexplored.

**Objective:**

This study aimed to explore the predictive power of OAS-Positive, its relationships with subjective well-being, and possible personality mediators related to positive functioning. We hypothesized that the abilities to feel grateful and savor positive life events mediate the relationship between optimistic thinking about positive outcomes and subjective well-being.

**Design:**

A cross-sectional design was implemented. The participants were 271 adults from Moscow and Moscow Region (M age = 32.42, SD=12.9).

**Results:**

The results of regression analysis showed that both life satisfaction and subjective happiness depended on gratitude, self-esteem, and dispositional optimism, but only happiness was predicted by savoring the moment. The results of structural equation modeling were consistent with the hypothesis since the structural model revealed that the effects of OAS-Positive on subjective well-being were fully mediated by gratitude and savoring the moment, as well as self-esteem and dispositional optimism. The mediated effects of OAS-Negative through self-esteem and gratitude were inconsistent, and its total indirect effect on subjective well-being was not significant.

**Conclusion:**

This research provides preliminary evidence that optimistic thinking about positive life events promotes subjective well-being through a system of positive psychological traits and attitudes which include gratitude and savoring the moment.

## Introduction

### Optimistic Attributional Style for Negative and Positive Events

The notion of attributional (or explanatory) style is a key concept of reformulated learned helplessness theory (Abramson, Seligman, & Teasdale, 1978) and its later version, the theory of hopelessness (Abramson, Metalsky, & Alloy, 1989). Attributional style is a cognitive personality variable that reflects the specific way people explain the causes of positive or negative events in their lives. It was originally conceptualized as having three dimensions: stability, globality, and internality (locus) (Peterson et al., 1982). It was assumed that people with an optimistic attributional style would tend to explain positive events with causes which are stable in time (*i.e.*, will always exist), global (*i.e.,* affecting all parts of their life — professional and private), and internal (due to them). On the contrary, optimists explain negative events with unstable (*i.e.*, this cause will never arise again), local (affecting just one specific part of their lives), and external causes (not due to them).

Conversely, the theory posited that people with a pessimistic attributional style would tend to explain negative events using stable, global, and internal causes, and positive events using unstable, local, and external causes. However, the locus dimension has been shown to demonstrate low reliability (Cutrona, Russell, & Jones, 1984; Smith, Caputi, & Crittenden, 2013), as well as questionable construct validity (Travers, Creed, & Morrissey, 2015). Consequently, as recommended by Abramson, Metalsky, and Alloy (1989), and Seligman (2002), many researchers have abandoned the locus dimension (*e.g.,* Houston, 1994).

Initially, most attributional style (AS) research was focused on the relationship between a pessimistic AS for negative events, and depression and ill-being. This approach was based on the Peterson’s idea that an AS for bad events is more informative than an AS for good events, because people’s reactions to negative uncontrollable life events conform to the learned helplessness theory and the theory of hopelessness (Peterson, 1991). Also, the early works of Seligman, Abramson, Semmel, and Baeyer (1979) showed that the association of a pessimistic AS for positive events with depression was weaker than the association of a pessimistic AS for negative events. The stronger association of a pessimistic AS for negative events than for positive events was also confirmed by a meta-analysis by Sweeney, Anderson, and Bailey (1986). Following these findings, many authors excluded positive situations from AS questionnaires (EASQ; Peterson & Villanova, 1988; Dykema, Bergbower, Doctora, & Peterson, 1996).

Studies by Peterson and several meta-analyses (Peterson, Villanova, & Raps, 1985; Sweeney et al., 1986; Hu, Zhang, & Yang, 2015) confirmed that a pessimistic attributional style for negative life outcomes is a reliable predictor of depression. Other studies revealed that a pessimistic attributional style for negative events is associated with anxiety (Lynd-Stevenson & Rigano, 1996; Ralph & Mineka, 1998), hostility (Boman, Smith, & Curtis, 2003), and neuroticism (Cheng & Furnham, 2001), as well as health issues (Peterson & Seligman, 1987; Yuan & Wang, 2016), and health complaints (Reilley, Geers, Lindsay, Deronde, & Dember, 2005). Studies of OAS-Positive have long been neglected, and Peterson would later conclude that this neglect of positive events was a regrettable mistake (Peterson & Park, 2007).

Factor analytic studies have proven the independence of positive and negative attributional style factors (Peterson, 1991; Xenikou et al., 1997), thus confirming the inefficiency of calculating the total score for attributional style and the importance of an OAS-Positive. Therefore, the recent trend of research in this area has switched from studies of OAS-Negative to analysis of the role of positive events attributions in people’s sense of well-being.

Studies of OAS-Positive have shown its positive relationship with subjective well-being (SWB), including happiness (Cheng & Furnham, 2001, 2003; Gordeeva & Osin, 2011) and life satisfaction (Rigby & Huebner, 2005), as well as extraversion and emotional stability (Rigby & Huebner, 2005), negative associations with depression (Gordeeva & Osin, 2011), and successful academic performance (Gordeeva et al., 2020). As for its relationship with various personality traits, Cheng and Furnham (2001) have shown that an OAS-positive correlated positively with extraversion, and was unrelated to neuroticism and psychoticism. In a non-clinical sample of adolescents, an optimistic attributional style for positive events moderated the relationship between negative life events and follow-up depressive symptoms (Vines & Nixon, 2009). However, to the best of our knowledge, all the studies that explored the relationship between an OAS-Positive and subjective well-being have been based on samples of adolescents and university students. Given the variation of attributions by age (Blanchard-Fields & Beatty, 2005), it is important to address a wider age range in future research.

Thus, an optimistic attributional style for positive events and an optimistic attributional style for negative events are two separate constructs, each of which has its own consequences. This study was dedicated to an OAS-Positive and its relationships with subjective well-being, since the mechanisms and potential mediators of attributions of positive events influencing people’s well-being and positive functioning remain underexplored. In particular, this study aimed to investigate a cognitive mediation model, in which selected personality traits that characterize positive personality functioning were expected to mediate the relationship between optimistic attributions and SWB.

### Looking for Personality Mediators of OAS-Positive and SWB

Our study focused on two well-known candidates for personality mediators — dispositional optimism and self-esteem — and two relatively new positive personality variables — gratitude and savoring — all of which imply noticing and valuing positive events. We hypothesized that these four variables may serve as mediators between an optimistic attributional style and well-being. All four variables have well-established relationships with well-being; however, gratitude and savoring, unlike dispositional optimism and self-esteem, have not been studied in relation to optimistic attributional style. Below we consider each personality variable and its relationships with positive functioning.

#### Gratitude

Theoretically, gratitude can be seen as an emotion, “an emotional response to a gift ” (Emmons, 2005, p. 239), or as a personality trait which is “part of a wider life orientation towards noticing and appreciating the positive in the world” (Wood, Froh, & Geraghty, 2010, p. 891). In our study, we follow Wood’s “life orientation” concept of gratitude. A large number of empirical studies have confirmed associations between gratitude and well-being: grateful people tend to be happier (Watkins, Van Gelder, & Frias, 2009; Wood et al., 2010), and have both a higher level of life satisfaction and a higher level of positive emotions over negative ones (Emmons & McCullough, 2003; Peterson, Ruch, Beerman, Park, & Seligman, 2007). Studies by Wood, Joseph, and Maltby (2008, 2009) have shown that gratitude was a reliable predictor of psychological well-being. The first study with a Russian-speaking sample confirmed the positive role of gratitude in well-being, a positive association between gratitude, self-esteem, and resilience, and a negative association between gratitude, depression, and interpersonal problems (Nartova-Bochaver & Kislitsa, 2017). Recent meta-analytic research suggests that gratitude interventions designed to increase appreciation of positive qualities, situations, and people in one’s life may improve psychological well-being, decreasing symptoms of depression and anxiety (Cregg & Cheavens, 2020).

The relationship between attributions for positive events and gratitude has many grounds, since optimistic thinking can facilitate a grateful disposition toward other people, which in turn will increase subjective well-being. In line with this idea, McCullough, Emmons, and Tsang (2002) argue that “attributions are central to gratitude, and attributional style may be central to the disposition toward gratitude” (p. 113). Indeed, gratitude was found to be a significant predictor of reduced depressive attributions (Ali & Rizwan, 2018). We suggest that dispositional gratitude could be based on the tendency of grateful people to attribute the reasons for success to the stable and reliable help of others.

#### Savoring

Although a much less studied topic, savoring has been found to play an important role in human well-being. The concept of savoring was introduced by Bryant and Veroff, who defined it as people’s “capacities to attend to, appreciate, and enhance the positive experiences in their lives” (Bryant & Veroff, 2007, p. 2). Savoring is not a process of experiencing a positive emotion; it is a cognitive process of directing attention to amplify and prolong positive emotions. In other words, savoring is a cognitive ability to stop and “smell the roses.” Bryant (2003) identifies three aspects of savoring: anticipating; savoring the moment; and reminiscing about past positive emotions or situations. These three kinds of savoring beliefs involve different temporal orientations to positive experience: perceived savoring capacity may stem from beliefs about one’s ability to derive pleasure in the present by savoring the moment, and intensifying or prolonging their positive feelings through specific thoughts and behaviors, but also by anticipating future positive events or by reminiscing about past positive events.

Bryant has shown that savoring beliefs were positively correlated with well-being, affect intensity, life satisfaction, and the intensity and frequency of happiness, as well as with aspects of positive functioning, like optimism, self-esteem, extraversion, internal locus of control, self-control, and reported self-control behaviors. They were negatively correlated with guilt, physical and social anhedonia, hopelessness, depression, neuroticism, and the frequency of unhappy and neutral affect, and uncorrelated with socially desirable responses (Bryant, 2003). Other studies have confirmed that savoring is associated with a wide range of variables reflecting positive functioning, such as optimism, internal locus of control, and self-control, as well as life satisfaction (Bryant, Smart, & King, 2005; Quoidbach, Berry, Hansenne, & Mikolajcza, 2010). Watson (2019) suggested that the inability to savor the pleasure of the obtained object could boost hedonic adaptation (Lyubomirsky, 2011), which leads to the aspiration to possess more and more. Research by Watson (2019) showed that savoring the moment was negatively associated with materialism, which in turn was related to lower levels of subjective well-being (Dittmar, Bond, Hurst, & Kasser, 2014). In a daily diary study which used experience sampling methodology, the multilevel modelling analyses confirmed that savoring is an important mechanism through which people derive happiness from positive events. In particular, momentary savoring both mediated and moderated the impact of daily positive events on a momentary happy mood (Jose, Lim, & Bryant, 2012). In our study we drew on these results and also on the Bryant and Veroff(2007)’s idea that savoring can serve as a mediator in the relationship between positive life-outcome and happiness.

#### Self-esteem

Self-esteem is an individual’s subjective evaluation of their own worth. High self-esteem has a strong relationship to well-being. According to Diener’s review (2009), positive association between self-esteem and well-being was confirmed in 11 studies. Later it was found that self-esteem was the most powerful predictor of happiness (Furnham & Cheng, 2000; Baumeister, Campbell, Krueger, & Vohs, 2003; Gordeeva & Osin, 2011). As to relationships between optimistic AS and self-esteem, it was shown that both types of optimistic attributional style — OAS-Positive and OAS-Negative — positively correlated with high self-esteem (Gordeeva & Osin, 2011).

#### Dispositional optimism

Dispositional optimism refers to generalized expectations regarding future outcomes: optimistic people believe that good things, rather than bad things, will happen (Carver & Scheier, 2014). According to Carver and Scheier, dispositional optimism relates to motivation: optimists exert effort, whereas pessimists disengage from effort. The relationship between dispositional optimism and well-being has been confirmed in a wide range of studies: optimists compared to pessimists are happier, and their level of satisfaction with life is higher (*e.g.*, Carver, Scheier, & Segerstrom, 2010; Mens, Scheier, & Carver, 2016). Optimists also show lower levels of anxiety and depression; have better health; use active coping strategies more often; and report better relationships with others (Carver et al., 2010).

On the other hand, dispositional optimism and optimistic attributional style demonstrate a low to moderate correlation (Reilley et al., 2005; Gordeeva, Sychev, Osin, & Titova Grandchamp, 2019). The similarities and specificities of the two types of optimism, as they are often called (Compton & Hoffman, 2019), were analyzed by Gordeeva, Sychev, and Osin (2017). It was shown that while these concepts are related, they differ in their mechanisms of interaction with well-being and academic performance.

Our study aimed to examine the relationship between OAS-positive and OAS-negative and subjective well-being, taking into account the role of gratitude, savoring, self-esteem, and dispositional optimism as possible mediators in these relationships. We hypothesized that an optimistic attributional style for positive events would be a significant predictor of life satisfaction and subjective happiness, and that this association is mediated by positive personality traits reflecting positive functioning.

In our study we used two well-established types of well-being variables — subjective happiness and satisfaction with life. According to Diener, subjective well-being is the scientific term for happiness and life satisfaction (2021). Lyubomirsky (2007) has described happiness as the “experience of joy, contentment, or positive well-being, combined with a sense that one’s life is good, meaningful, and worthwhile” (p. 32). Life satisfaction involves a favorable attitude towards one’s life rather than an assessment of current feelings; it is a measure of well-being assessed in terms of satisfaction with relationships, achieved goals, and self-perceived ability to cope with one’s daily life (Diener, 2021). An individual’s levels of subjective well-being are influenced by both internal and external factors; this study concentrated on the former and explored the importance of cognitive variables in the processes that underlie SWB.

## Methods

### Participants

The participants were 271 adults from Moscow and the Moscow Region, of whom 41% were university students and 59% were employees working in the public and private sectors. The sample comprised 238 (88%) women and 33 (12%) men; M age = 32.42, SD = 12.9, age range 18–78 years.

### Measures

To measure *optimistic attributional style* as a stable trait and a possible predictor of subjective well-being, we used a modified version of the Attributional Style Questionnaire (Peterson et al., 1982), which featured 10 achievement situations (five positive and five negative) (Gordeeva et al., 2019). A sample negative scenario was: “You have received negative feedback from a respected colleague.” Participants were instructed to imagine that each situation had actually happened to them, to write down its most likely cause, and then rate this cause using a 6-point Likert-type scale on two main dimensions of attributional style: stability (this cause will never happen again or will always be present) and globality (this cause influences just this particular situation or influences all situations in my life). An optimistic attributional style for explaining positive events (OAS-Positive) score was computed by summing the stability and globality ratings for positive situations, and an optimistic AS for negative events (OAS-Negative) score was computed by first reversing the ratings of the negative situations, and then summing them. The reliability coefficients of all the scales used in this study are presented in *Table 1.*

**Table 1:** Descriptive statistics and correlations between optimistic attributional style, subjective well-being and personality variables

	*α*	Mean	SD	1	2	3	4	5	6	7	8	9	10
1. Satisfaction with life	.81	4.74	1.06	—									
2. Subjective happiness	.81	4.91	1.23	.66***	—								
3. OAS-Positive	.80	4.38	.95	.35***	.35***	—							
4. OAS-Negative	.85	4.21	1.01	.02	.14**	.01	—						
5. Dispositional optimism	.89	2.99	.79	.45***	.65***	.32***	.15**	—					
6. Self-esteem	.83	3.13	.50	.43***	.61***	.35***	.30***	.59***	—				
7. Gratitude	.73	5.78	.91	.43***	.47***	.29***	–.01	.40***	.32***	—			
8. Savoring, anticipating	.85	5.31	1.03	.13*	.26***	.18**	.01	.37***	.25***	.27***	—		
9. Savoring the moment	.82	4.96	1.11	.40***	.64***	.24***	.16*	.59***	.52***	.43***	.45***	—	
10. Savoring, reminiscing	.84	5.45	1.01	.28***	.35***	.15*	.07	.47***	.30***	.35***	.56***	.59***	—
11. Savoring, Total	.91	5.24	.87	.33***	.51***	.23***	.09	.57***	.44***	.42***	.80***	.83***	.86***

Note. Pairwise deletion of missing data (N is from 261 to 268), * = p ≤ .05; ** = p ≤ .01; *** = p ≤ .001; α = Cronbach’s.

*Savoring* was measured by the Russian version of the Savoring Beliefs Inventory (SBI) (Bryant, 2003), which was developed specifically for this study; direct and back translation of the questionnaire was implemented by two bilingual experts. The questionnaire consisted of 24 items that constituted three scales: 1) savoring the moment; 2) anticipating; and 3) reminiscing. Each scale consisted of eight items, half of which were worded positively (*e.g.,* “I know how to make the most of a good time”) and the other half were worded negatively (*e.g.,* “When it comes to enjoying myself, I’m my own ‘worst enemy’”). Respondents rated their agreement with each item using a 7-point Likert scale. The total scale reliability measured by Cronbach’s α was 0.91, and the reliability of the subscales varied from 0.82 to 0.85, which was considered satisfactory.

To assess *gratitude,* we used the Russian version of GQ-6 (McCullough et al., 2002), which was developed for this study. Direct and back translation of the questionnaire was implemented by two bilingual experts. The original version consisted of six items, with four positively worded statements (*e.g.,* “I have so much in life to be thankful for”) and two negatively worded statements (*e.g.,* “When I look at the world, I don’t see much to be grateful for”) to be rated on a 7-point Likert scale. In the Russian version of the questionnaire, the reverse items showed weak consistency with the positively worded ones (which is quite a common phenomenon, see Suarez-Alvarez et al., 2018). To improve scale reliability, it was decided to exclude the two reverse items and use four-item version which demonstrated satisfactory reliability (Cronbach’s α = .73).

*Dispositional optimism* was assessed by the Russian version of the Life Orientation Test (Scheier, Carver, & Bridges, 1994; Gordeeva, Sychev, & Osin, 2010). This instrument included four positively worded items, four negatively worded items, and four filler items rated on 4-point Likert scale (Cronbach’s α = .89).

*Self-Esteem* was assessed using the Russian version of the Rosenberg Self-Esteem Scale (Rosenberg, 1965; Bodalev & Stolin, 1987). The scale consisted of 10 items, five positively and five negatively worded, to be rated on a 4-point Likert scale (Cronbach’s α = .83).

*Subjective well-being. Life satisfaction and happiness* were measured with Russian versions (Osin & Leontiev, 2020) of the Satisfaction with Life Scale (SWLS) (Diener, Emmons, Larsen, & Griffin, 1985) and the Subjective Happiness Scale (SHS) (Lyubomirsky & Lepper, 1999). The SWLS consisted of five items which were to be rated on a 5-point Likert scale, and the SHS consisted of four items to be rated on a 7-point Likert scale (Cronbach’s α for both scales in this study was .81).

### Procedure

This research was introduced as a study conducted by Psychology Department of Lomonosov Moscow State University entitled “Study of the sources of happiness and psychological well-being.” We asked participants to help science by completing a battery of tests. As a reward we offered individual feedback on their personality “happiness profile.” Confidentiality was stressed. Most participants (N = 171) completed the online version of the survey.

Since this group was dominated by young respondents (average age M = 28.37, SD = 9.12), to increase the representativeness of the sample, a paper survey was conducted among more mature and elderly people (M = 39.36, SD = 15.29), represented mainly by teachers and other staffat two Moscow schools. The effects of which survey type was used were analyzed. The revealed effects were quite weak and did not affect the main assumed predictors (OAS) and dependent variables (well-being indicators). Thus, we concluded that the joint analysis of the “online” and “paper” groups did not compromise the validity of the research findings.

### Data Analysis

The structural equation modeling was undertaken in Mplus 8, using a robust maximum likelihood estimation (Muthen & Muthen, 2015). A full-information maximum likelihood method (Enders & Bandalos, 2001) was used to analyze missing data (10 cases, 3.7% of the sample). To assess the significance of mediated effects in the structural model, a bootstrap analysis with 5000 samples was carried out in Mplus (Wang & Wang, 2019). Other analyses, including descriptive statistics, correlations, regression analysis, and t-tests were carried out using SPSS.

## Results

The correlations among the study variables presented in *Table 1* showed that subjective happiness was related to all other measures, including both indicators of OAS, and all indicators of savoring, dispositional optimism, self-esteem, and gratitude. Life satisfaction was correlated with all measured variables with the exception of OAS-Negative. An OAS-Positive also demonstrated significant correlations with all other variables, however, it was not associated with an OAS-Negative, while the latter showed significant correlations with only three variables: self-esteem, dispositional optimism, and savoring the moment. All scales of savoring, dispositional optimism, and self-esteem were moderately or strongly interrelated (*see Table 1*).

Age showed moderate correlations with an OAS-Negative (r = .36; p * .001), self-esteem (r = .25; p ≤ .001), gratitude (r = .17; p ≤ .01), subjective happiness (r = .13; p ≤ .05), and savoring the moment (r = .13; p ≤ .05). These results indicated that age covariates with many study variables, including indicators of an OAS and well-being, so measures should be taken in further analyses to control its effects. Analysis of sex differences using the Student’s t-test revealed that the women had lower mean scores of an OAS-Positive (M(women) = 4.34, M(men) = 4.71; t(265) = 2.10; p ≤ .05) and higher mean scores of SBI-Future (M(women) = 5.36, M(men) = 4.98; t(264) = 1.97; p ≤ .05).

We then applied regression analysis to estimate the relationships between indicators of well-being and the set of its potential predictors which included dispositional optimism, self-esteem, gratitude, and savoring, controlling for age. The results of this analysis (*Table 2*) revealed that life satisfaction was positively related to an OAS-Positive, dispositional optimism, self-esteem, and gratitude. Savoring the future showed a relatively small negative effect on life satisfaction. Happiness was positively associated with dispositional optimism, self-esteem, gratitude, and savoring the moment. These results confirmed the positive effect of an OAS-Positive on subjective wellbeing, but this effect may be direct or mediated by some other variables.

**Table 2:** Linear regression models for Life satisfaction and Subjective happiness (N=261)

Predictors	Dependent variables			
	Life satisfaction		Subjective happiness	
	β	t(251)	β	t(251)
OAS-Positive	0.13*	2.29	0.07	1.48
OAS-Negative	–0.05	–0.82	–0.01	–0.33
Dispositional optimism	0.17*	2.38	0.29***	5.08
Self-esteem	0.22**	3.25	0.24***	4.42
Gratitude	0.25***	4.25	0.17***	3.50
Savoring, anticipating	–0.16*	–2.52	–0.06	–1.30
Savoring the moment	0.13	1.71	0.35***	5.96
Savoring, reminiscing	0.04	0.56	–0.09	–1.67
Age	–0.11	–1.92	–0.03	–0.64
*R* ^2^	0.37		0.59	
F(9,251)	16.53		42.03	
p-level	≤ 0.001		≤ 0.001	

Note. β — standardized regression coefficients, * = p≤.05; ** = p≤.01; *** = p≤.001.

To test our hypothesis about mediated relations between an OAS and well-being, we applied structural equation modelling. Life satisfaction and subjective happiness were included in the model as dependent variables, along with an OAS-Positive, an OAS-Negative, and four potential mediators of the effect of OAS on well-being (all of them were allowed to correlate). The only savoring scale included in the model was the savoring-the-moment scale because of its highly significant positive effect on happiness in the regression analysis results. Given the results presented above, the participants’ age was added as a covariate of OAS-Negative and predictor of self-esteem, gratitude, savoring the moment, and subjective happiness. After removing all non-significant paths from this model, we obtained satisfactory fit: X^2^ = 27.10; df = 13; p = 0.012; CFI= 0.980; TLI = 0.949; SRMR = 0.056; RMSEA = 0.063 (90% CI = [0.029, 0.097]); PCLOSE = 0.230; N = 271.

Then we investigated modification indices and added a path from age to life satisfaction in the model. The final model presented in the figure below showed good fit: X^2^ = 19.75; df = 12; p = 0.072; CFI= 0.989; TLI = 0.969; SRMR = 0.055; RMSEA = 0.049 (90% CI = [0.000, 0.086]); PCLOSE = 0.474; N = 271.

**Figure 1.**
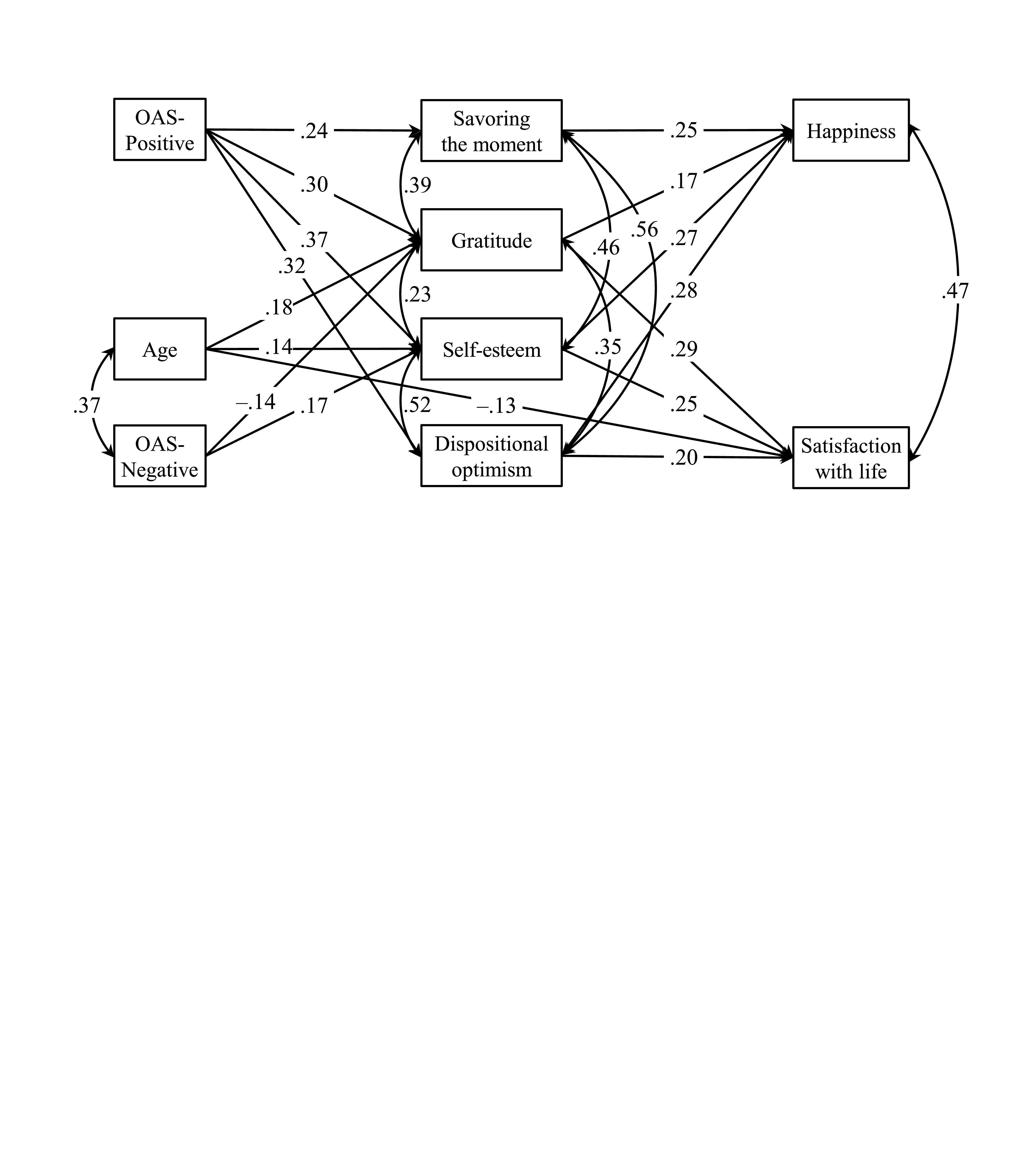
The structural model of relations between the two types of optimistic attributional style (OAS), two indicators of subjective well-being, and four mediators

*Note. All coefficients are standardized and significant at p ≤ .01; N = 271.*

The results of analyzing the indirect effects of an OAS and age on the subjective well-being indicators in the presented structural model revealed that all the effects mediated by individual mediators were statistically significant (*see Table 3*).

Thus, the structural model revealed that the effects of an OAS-Positive on subjective well-being were fully mediated by gratitude, savoring the moment, self-esteem, and dispositional optimism. Both life satisfaction and subjective happiness depended on gratitude, self-esteem, and dispositional optimism, but only happiness hinged on savoring the moment. The mediated effects of an OAS-Negative through self-esteem and gratitude were inconsistent, so its total indirect effect on subjective well-being was not significant.

**Table 3:** Indirect effects of optimistic attributional style (OAS) for positive and negative events on life satisfaction and subjective happiness.

Dependent variable	Predictors	Mediators	Standardized indirect effect	*p-level*
Satisfaction with life	OAS-Positive	All (Self-esteem, dispositional optimism, gratitude)	.24	*≤ .001*
Satisfaction with life	OAS-Positive	Self-esteem	.09	*≤ .01*
Satisfaction with life	OAS-Positive	Dispositional optimism	.06	*≤ .05*
Satisfaction with life	OAS-Positive	Gratitude	.09	*≤ .001*
Satisfaction with life	OAS-Negative	All (Self-esteem and gratitude)	.01	*n. s.*
Satisfaction with life	OAS-Negative	Self-esteem	.05	*≤ .05*
Satisfaction with life	OAS-Negative	Gratitude	–.04	*≤ .05*
Subjective happiness	OAS-Positive	All (Self-esteem, dispositional optimism, gratitude and savoring the moment)	.30	*≤ .001*
Subjective happiness	OAS-Positive	Self-esteem	.10	*≤ .001*
Subjective happiness	OAS-Positive	Dispositional optimism	.09	*≤ .001*
Subjective happiness	OAS-Positive	Gratitude	.05	*≤ .01*
Subjective happiness	OAS-Positive	Savoring the moment	.06	*≤ .01*
Subjective happiness	OAS-Negative	All (Self-esteem and gratitude)	.02	*n. s.*
Subjective happiness	OAS-Negative	Self-esteem	.05	*≤ .05*
Subjective happiness	OAS-Negative	Gratitude	–.02	*≤ .05*

## Discussion

DeNeve and Cooper (1998) hypothesized that “perhaps what is most critical to subjective well-being is not simply the tendency to experience positive or negative emotion, but the tendency to make either positive or negative attributions” (p. 219). From this standpoint, our study sought to investigate a cognitive mediation model, in which selected positive personality traits were expected to mediate the relationship between optimistic attributions and SWB. We have found that the optimistic attributional style for positive life events uniquely predicted subjective well-being, including happiness and life satisfaction, through positive personality traits such as gratitude, savoring the moment, dispositional optimism, and self-esteem. In contrast, an OAS for negative events did not predict either life satisfaction, or subjective happiness. Also, our results showed once again that the ability to explain the causes of positive events optimistically, *i.e.*, see them as global and stable, was unrelated to the ability to explain the causes of negative events as local and temporary; these are two different types of optimistic thinking.

Thus, this study confirmed previous results on the relationships between the trait of savoring and well-being (Bryant, 2003) and went further to establish the role of savoring the moment as a mediator between optimistic thinking about positive life outcomes and happiness. Moreover, the results of our study suggest that there are some differences in the predictive power of the scales. Savoring the moment was significantly more important for well-being, and especially subjective happiness, than savoring of past events and savoring possible future positive events. This may be due to the different mechanisms of savoring implied in these orientations, which thus need to be studied. For example, savoring the moment is rather close to mindfulness (Kiken, Lundberg, & Fredrickson, 2017; Watson, 2019), which is the ability to have a clear focus upon what is happening in the present moment, and involves intention, attention, and attitude.

The mediational role of gratitude and savoring the moment deserves further attention due to their joint, but also complimentary nature, since the former reflects a more eudaimonic perspective (Wood et al., 2010), while the latter reflects a more hedonic one (Diener, Lucas, Oishi, Hall, & Donnellan, 2018). Gratitude is closer to eudaimonic strategy of life, which is defined as the presence of personal and social skills and abilities that contribute to optimal psychosocial functioning (Ryff, 2018). With respect to the dispositional optimism and self-esteem findings, our results confirmed the hypothesis and previous results in this field.

The sex differences found in the study, and the finding that women had a lower mean rate of an OAS-Positive, were small and did not correspond to our previous results (Gordeeva et al., 2019), which showed no sex differences on this variable. This means that this part of the research should be replicated with a bigger sample of men. It was also found that women showed a higher level of anticipatory savoring than men, which can be explained by the reality that women often have hopes for a more favorable future associated with family life, and corresponds to the higher dispositional optimism which has been previously found in Russian women (Gordeeva, Sychev, & Osin, 2021).

The strength of this study was its nonstudent sample, which included adults of different ranges of age and professions. The positive relationship of age with an OAS-Negative, gratitude, savoring the moment, and self-esteem, and the negative one with life satisfaction, probably reflected the conflicting trends inherent in aging.

## Conclusion

Our results point to the conclusion that the ability to explain good events optimistically is unrelated to the ability to optimistically explain bad events, and that it’s the former that’s essential for individuals’ positive functioning and well-being. The cultivation of optimistic thinking promotes gratitude, a strategy that essentially involves appreciative positive attention, and savoring the moment, as well as feelings of self-worth and positive expectations about the future.

## Limitations

This study had some limitations, the most significant of which was associated with its cross-sectional nature. Despite the path model we presented, we are aware that the study’s cross-sectional design did not allow us to assess causality.

It is also important to note the limitation due to the sample not being balanced by sex, since the vast majority of participants were female (88%). This characteristic can constrain generalizability of the study’s findings. Given the sex differences in OAS, dispositional optimism, and savoring, it is important to confirm these findings using a sample more balanced by sex. At the same time it is important to note that the sex differences may be culture specific: for example, sex differences on savoring the present moment subscale (which showed to be the main predictor of happiness) were the smallest (Bryant, 2003) and in our sample were not significant.

Yet another limitation was the possible validity issues of the Russian version of GQ-6 scale, since it included only four items and did not include the two reverse items. At the same time since other researchers faced the same problems with these items (see Chen, Chen, Kee, & Tsai, 2009; Langer, Ulloa, Aguilar-Parra, Araya-Veliz, & Brito, 2016), the four items Russian gratitude measure was considered to be satisfactory enough for research purposes (Cronbach’s α = .73).

Finally, taking into account the role of culture in the relationship between gratitude and well-being (Peterson et al., 2007), further research on other cultural samples will be of interest.
